# Supplementary figures and images for: Electrical coupling controls dimensionality and chaotic firing of inferior olive neurons
Source: PLoS Comput Biol. 2020 Jul 30;16(7):e1008075. doi: 10.1371/journal.pcbi.1008075 (PMC7419012; doi:10.1371/journal.pcbi.1008075)

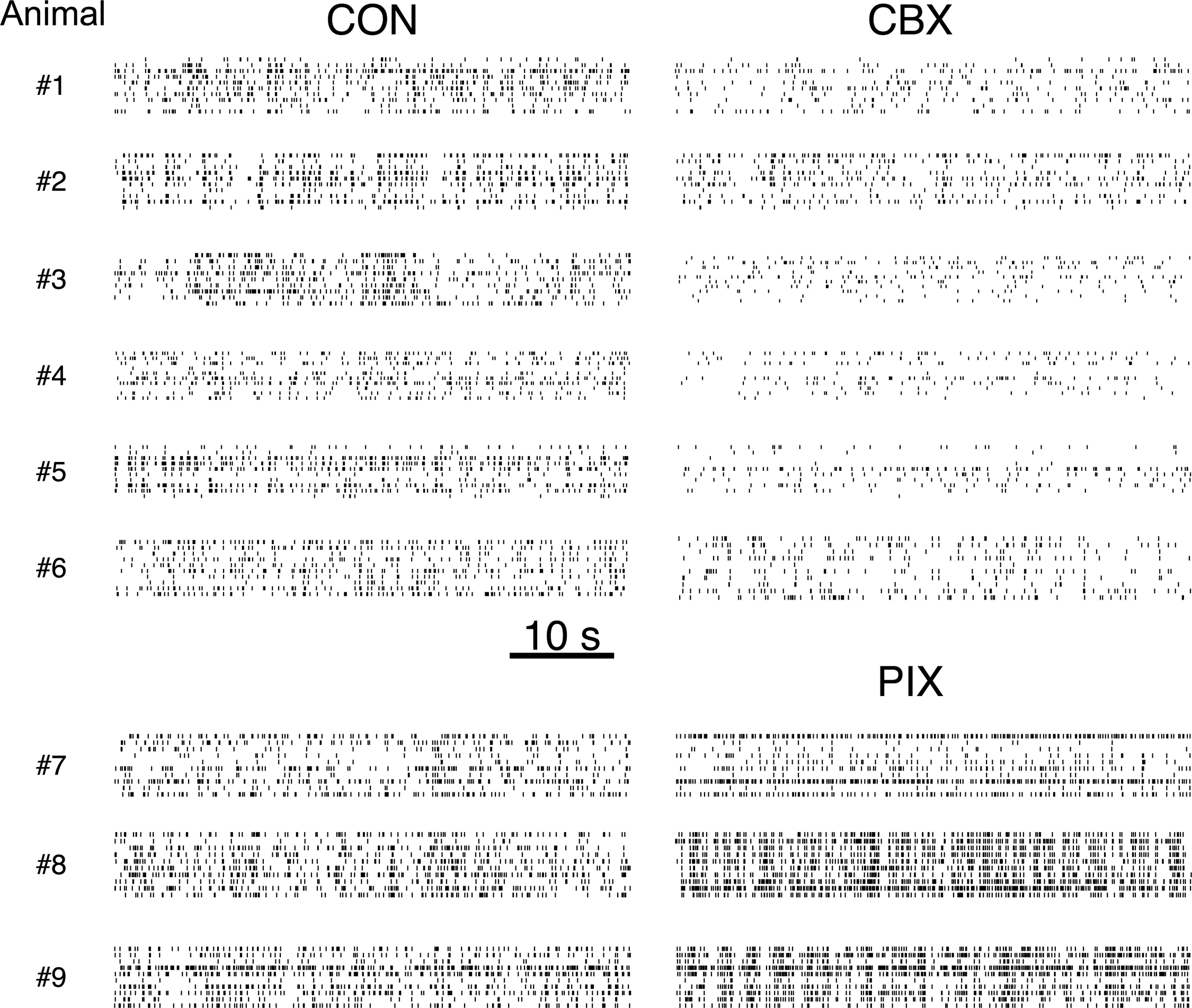

Supplement: S1 Fig — A: Spike data in 50 second of 10 representative neurons in 9 animals with the physiological conditions (CBX and PIX) in the right and the control condition (CON) in the left columns. (TIF) [file pcbi.1008075.s001.tif]

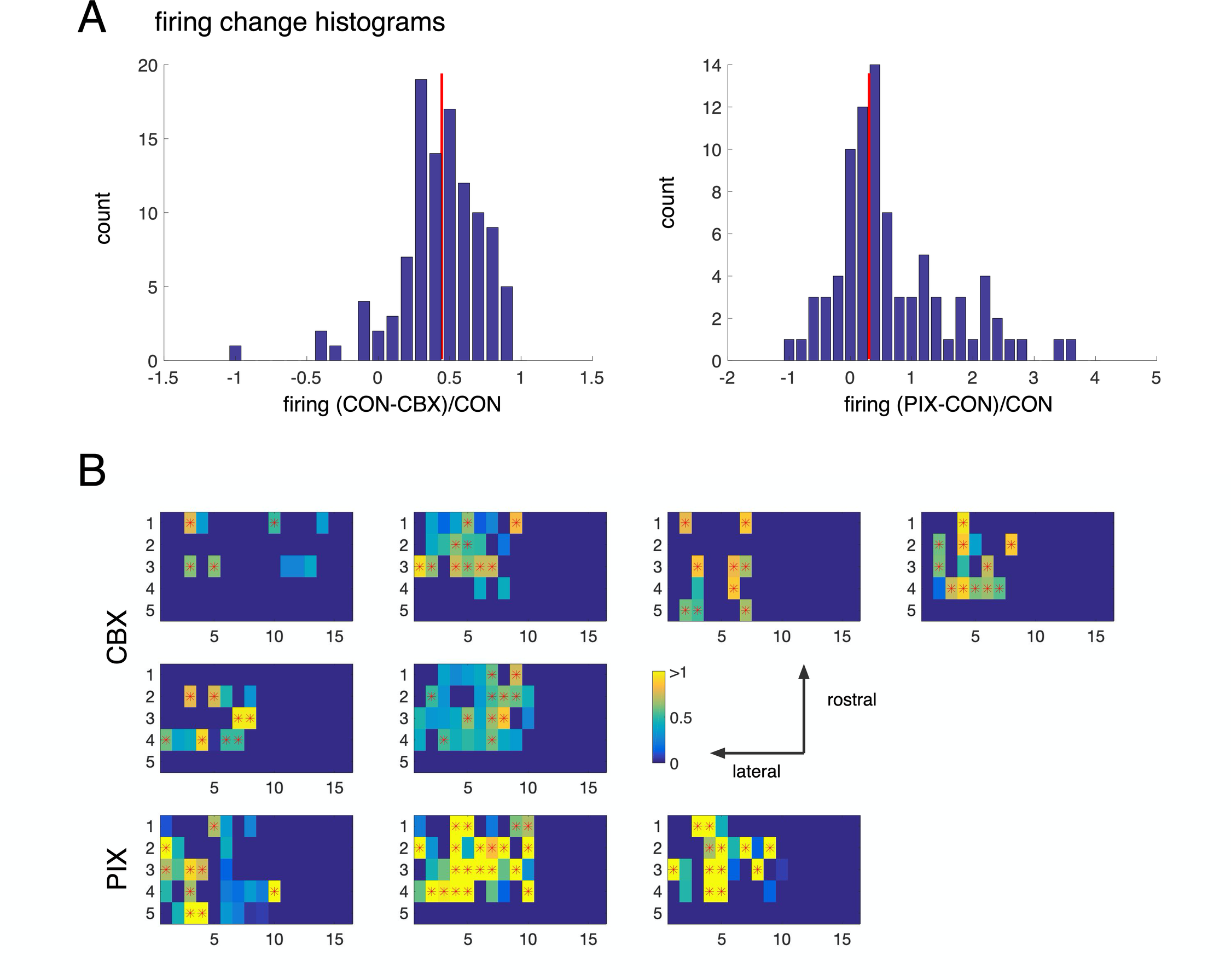

Supplement: S2 Fig — A: the histograms of firing rate change by drug treatments (decreased by CBX and increased by PIX) compared to the CON condition. The red lines indicate the thresholds (50%) for selecting the neurons for analysis. B: pseudo-color maps show the firing rate change by drug treatments of the neurons in the micro-electrode arrays for six CBX animals (top two rows) and three PIX animals (bottom row). Red asterisks indicated the selected neurons, whose firing rate changes exceed the thresholds. (TIF) [file pcbi.1008075.s002.tif]

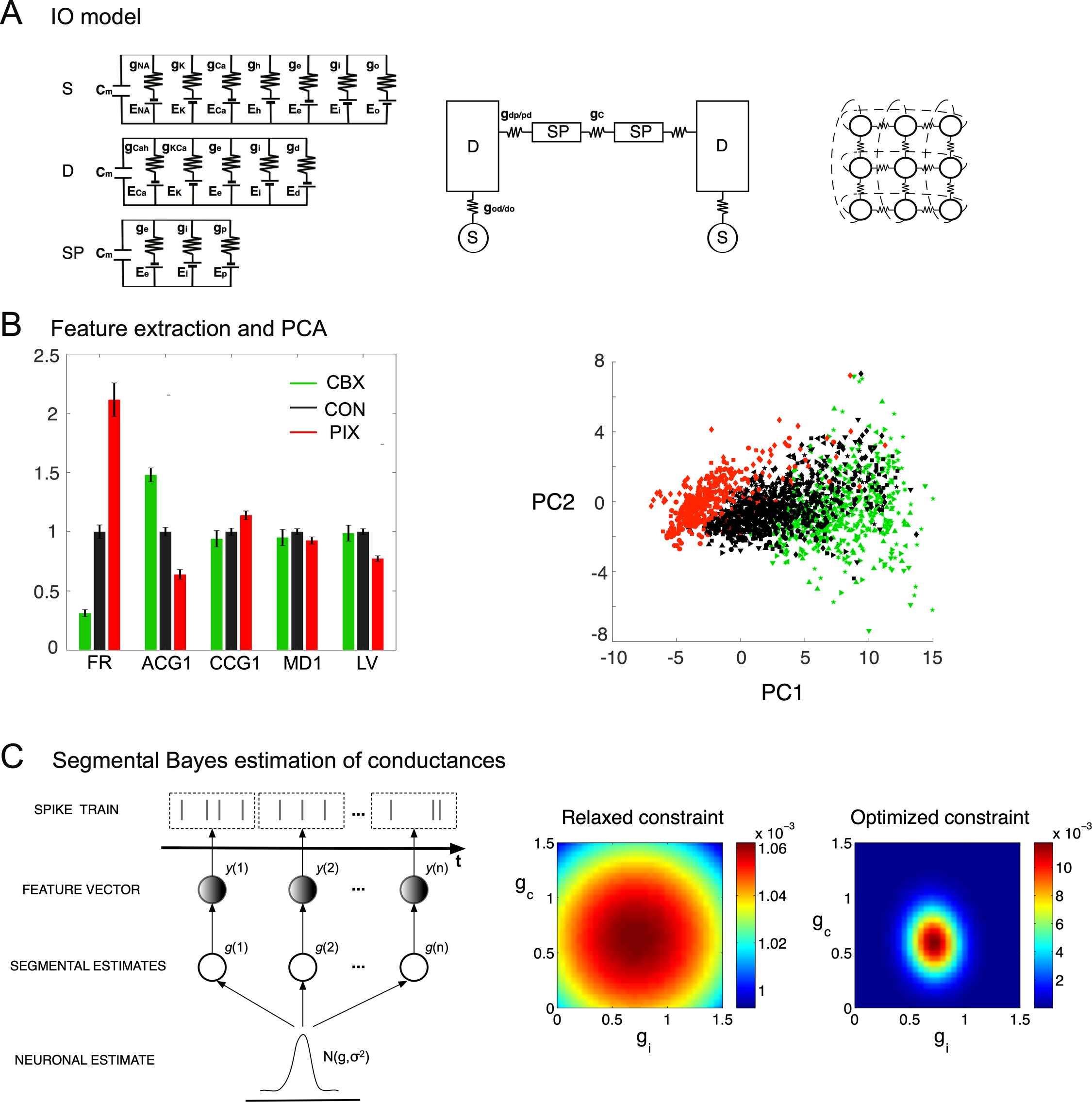

Supplement: S3 Fig — A: left, electrical circuit equivalents of the soma (S), dendrite (D) and spine compartments (SP) of a model IO neuron. middle, the connection of two IO neurons via a gap junctional conductance gc that connects the spine compartments. right, The IO network, which consists of 3x3 neurons, each of which is connected to its four neighboring neurons as shown. B: Left, five major features (FR = firing rate, ACG1 = auto-correlogram in 50 ms bin, CCG1 = cross-correlogram in 50 ms bin, MD1 = the first fraction of the minimal distance distribution, LV = local variation) extracted from spiking data of the three conditions (see [56] for detailed definitions of the features). Each feature was normalized by the mean value of the CON level. Right, the top two principal components of the extracted features. C: Flow chart of conductance estimation for each neuron. To account for highly non-stationary of the spike patterns in the three data condition, we divided the spike data of each neuron into small time-segments, applied the Bayesian inference to estimate gi and gc for every segment under the assumption that segmental estimates were drawn from a single neuronal estimate of a normal (Gaussian) distribution with unknown mean and the variance as a prior (left). The variance was optimized so as to maximize the fit of the data and the model in the PCA space. The posterior estimation for a representative neuron's gi and gc conductances (right). A broad probability distribution was found when the variance was relaxed but a much smaller distribution resulted when optimized variance was used [51]. (TIF) [file pcbi.1008075.s003.tif]

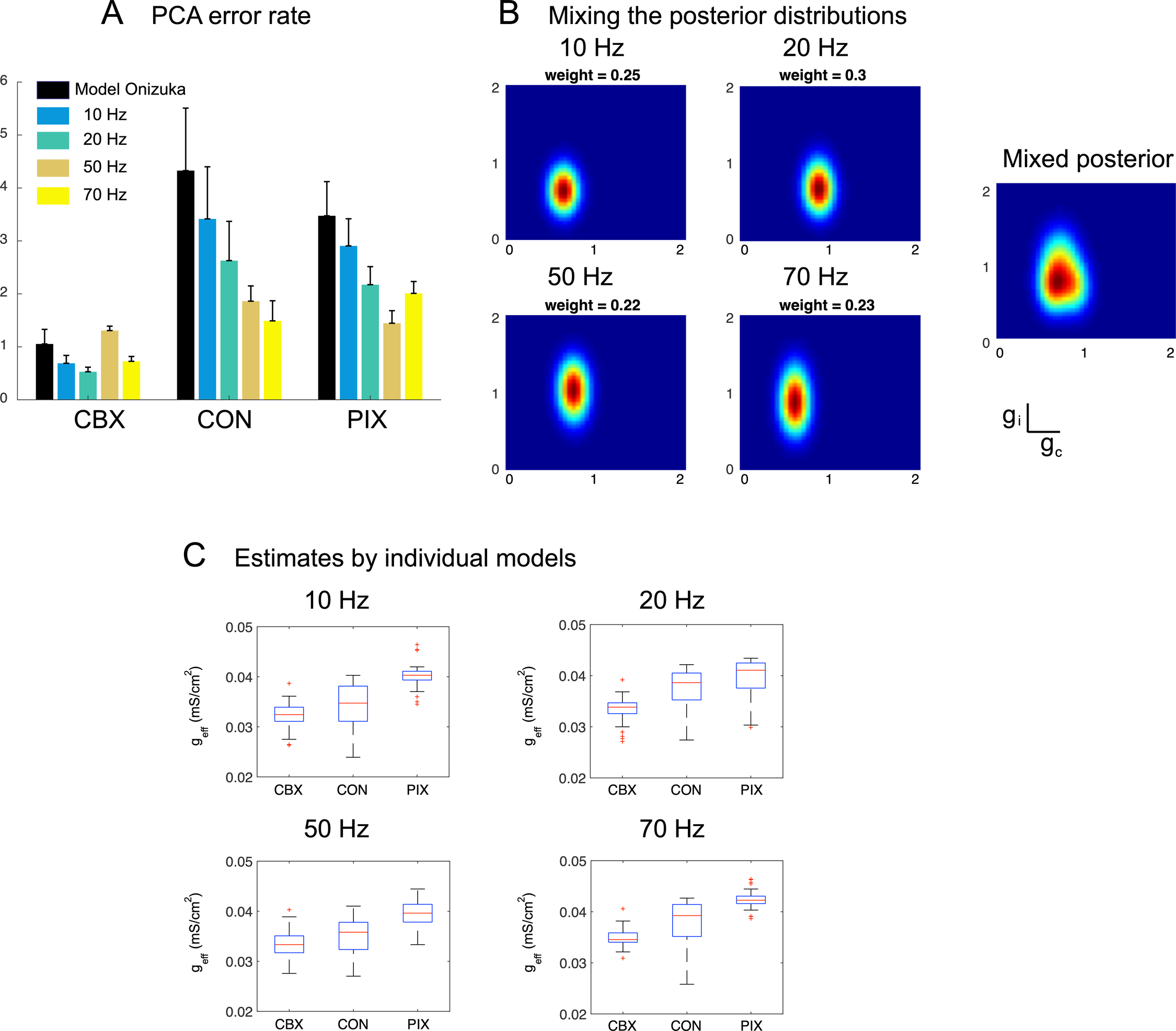

Supplement: S4 Fig — A: PCA error rates of the gi and gc estimates by the segmental Bayesian inference averaged for the entire IO neurons for CBX, CON, and PIX conditions for four different models (color bars) in comparison with the previous model (black bar, [56]). The error bars are of 95% CIs. B: Posterior probabilities of a representative IO neuron by individual models and the mixed posterior probability with the weights determined by the evidence of Bayesian inference. C: boxplots show estimates of the effective coupling geff of the three data conditions in the four individual models. (TIF) [file pcbi.1008075.s004.tif]

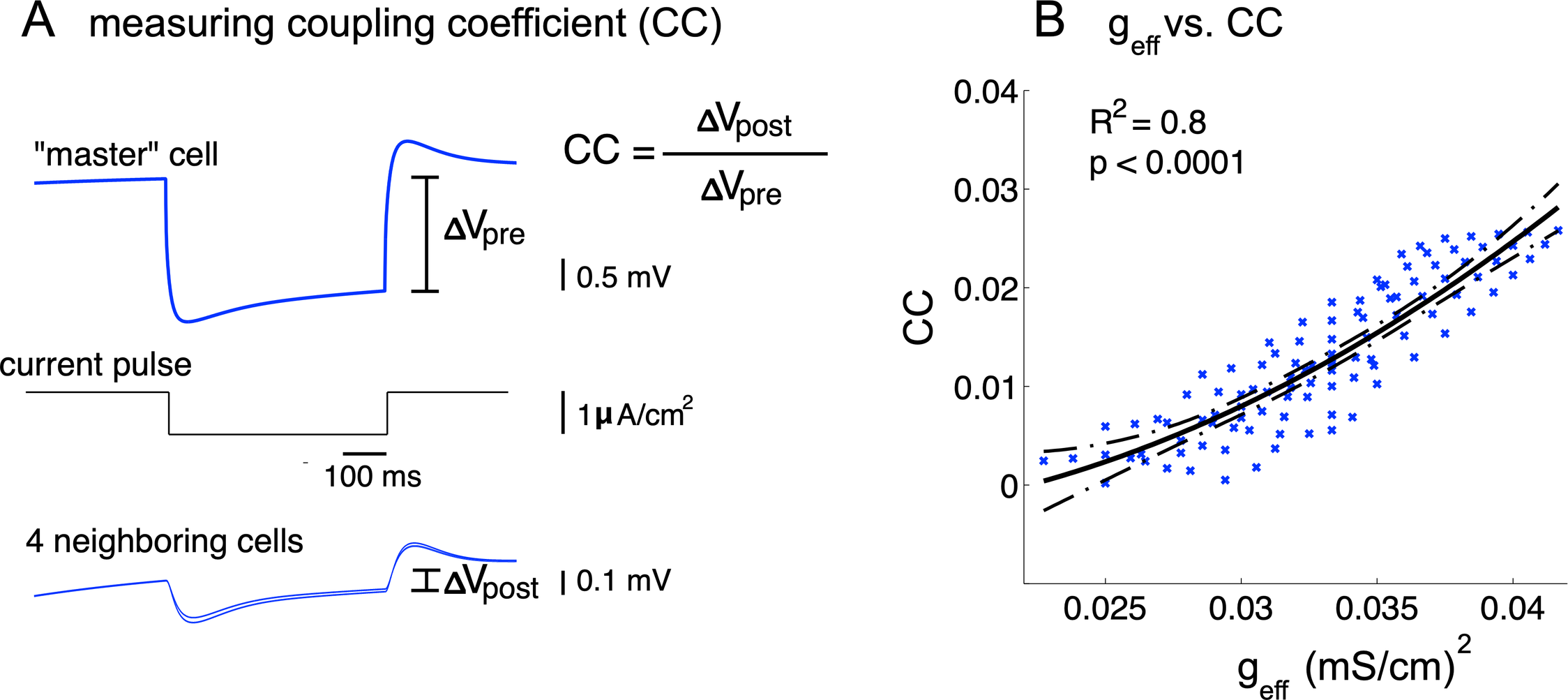

Supplement: S5 Fig — A: We injected a current pulse of -1 μA/cm2 to a cell and recorded the steady-state voltage change of this “master” cell and its four post-junctional cells. B: We computed the CCs for hundreds of gi and gc values in the range over which the estimated conductances of the data distributed, and found a strong positive correlation between the effective coupling and the CC (R2 = 0.8, p < 0.0001). Note that the non-linear fit represents the nature of deriving geff from gi and gc following Eq (1). (TIF) [file pcbi.1008075.s005.tif]

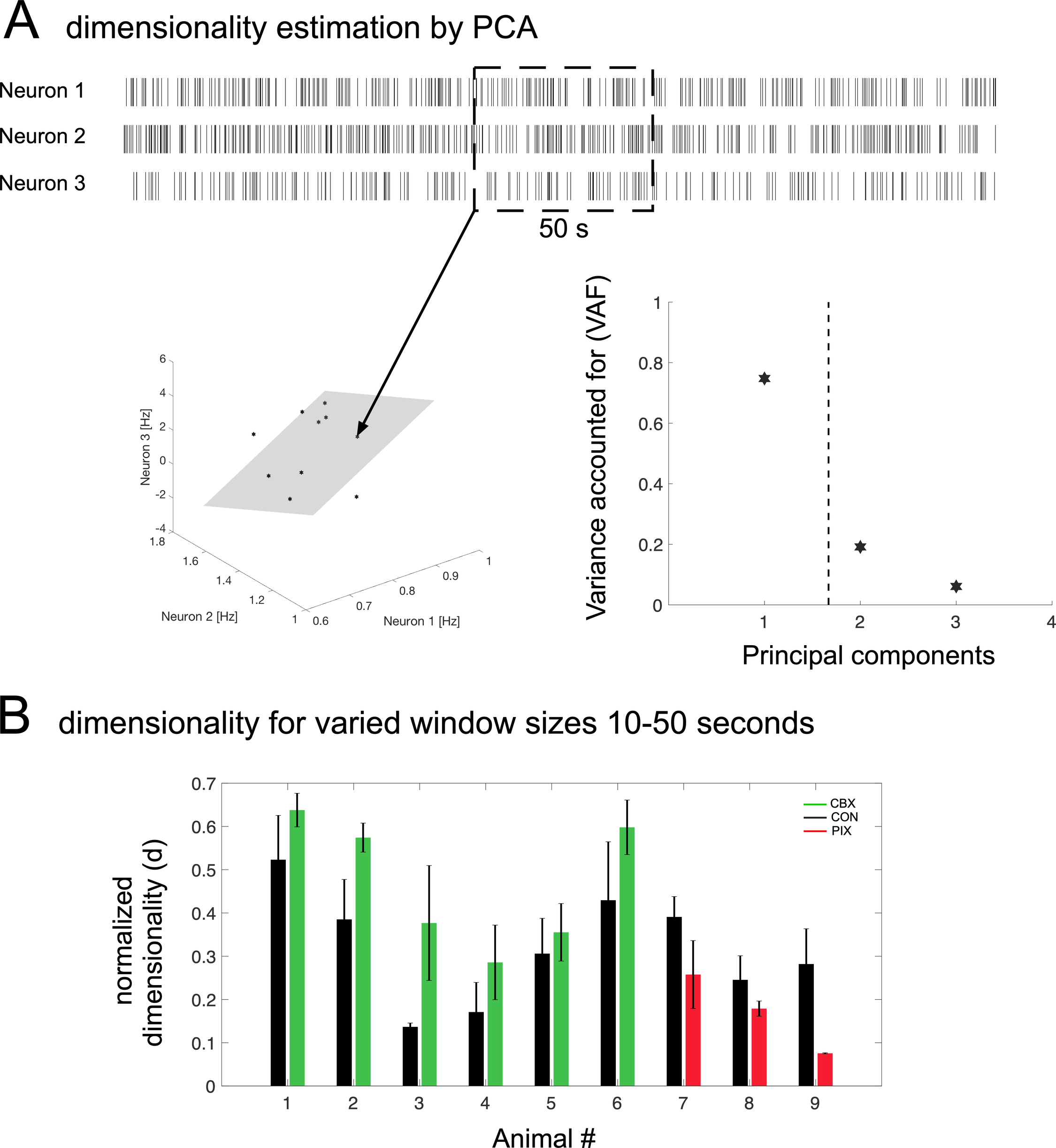

Supplement: S6 Fig — A: Illustration of the principal component analysis (PCA) for the firing rate vectors extracted from 50-second windows of three neurons of Animal #6 in the CON condition. The estimated dimensionality d = 1.86 (dashed dark line, Eq 5), indicates that the approximately 2-dimensional subspace (shaded gray plane) can explain more than 90% of the variance of neural firing dynamics. B: Estimating dimensionality (Eq 5) with varied window lengths from 10–50 seconds for 9 animals in the three data conditions showing the robustness of dimensionality estimation against the window length. The error bars are of 95% CIs. (TIF) [file pcbi.1008075.s006.tif]

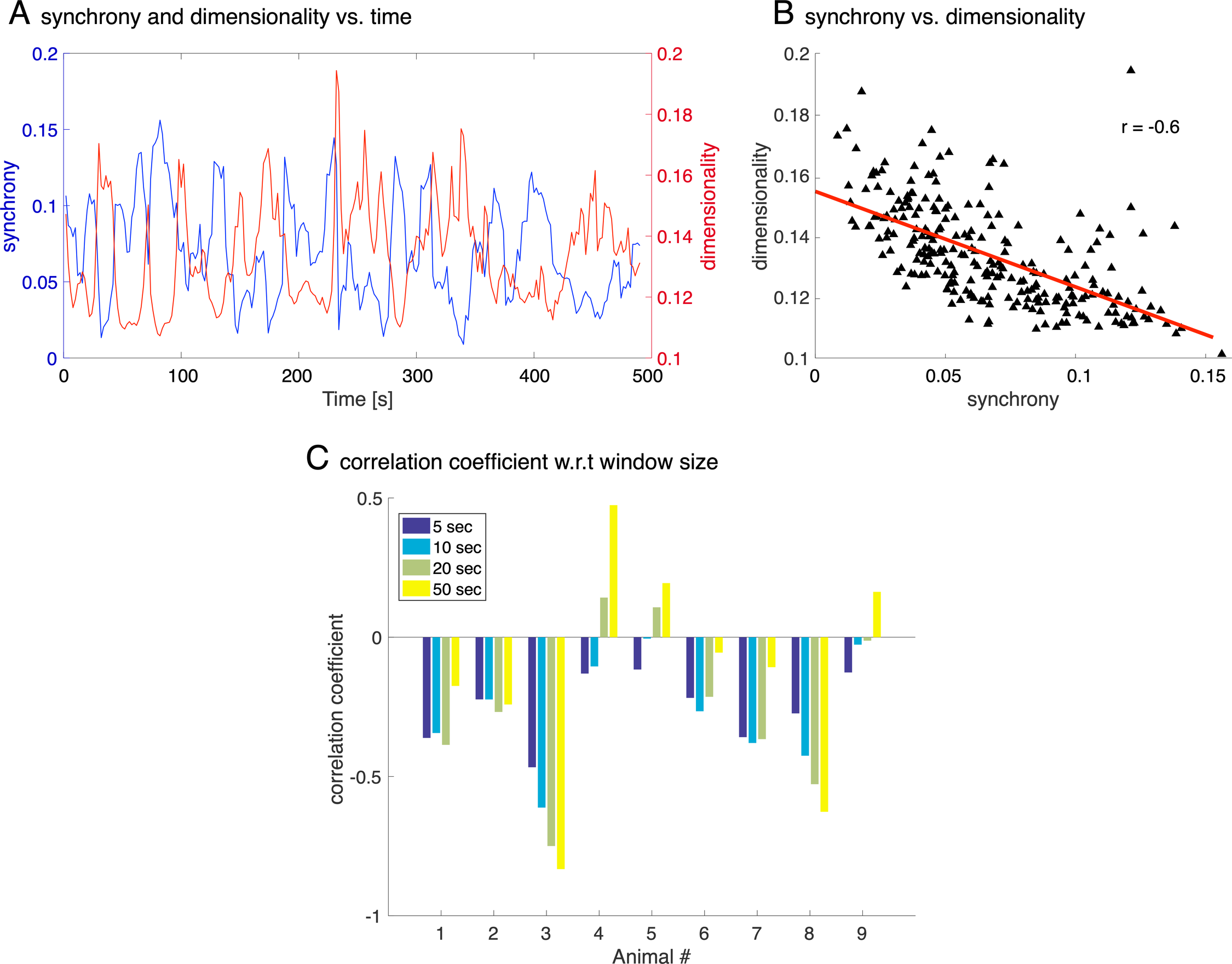

Supplement: S7 Fig — We first divided the complex spike data of 9 animals in the control condition into a series of shorter segments using a moving window of length 5, 10, 20, or 50 s, whose start incremented in 1 second steps. Next, we computed the dimensionality and the synchrony (both in 10 milli-second timescale) for each segment (A) and measured the correlation coefficient between those two metrics (B). The correlation coefficients were negative for almost all animals and window lengths (C). These results provide clear evidence that variations of synchrony within the physiological range are negatively correlated with changes in dimensionality. Note that the control data of spontaneous complex spike activity in our study is within the range of physiologically occurring synchrony levels. A: time-varying synchrony (left ordinate) and dimensionality (right ordinate) in successive, overlapped 10-s sliding windows of the control recording from animal #3. B: Scatterplot of correlation versus dimensionality values shown in A shows the presence of a significant negative correlation (r = -0.6). C: synchrony vs. dimensionality correlation coefficients for 9 control animals and all tested window lengths. (TIF) [file pcbi.1008075.s007.tif]

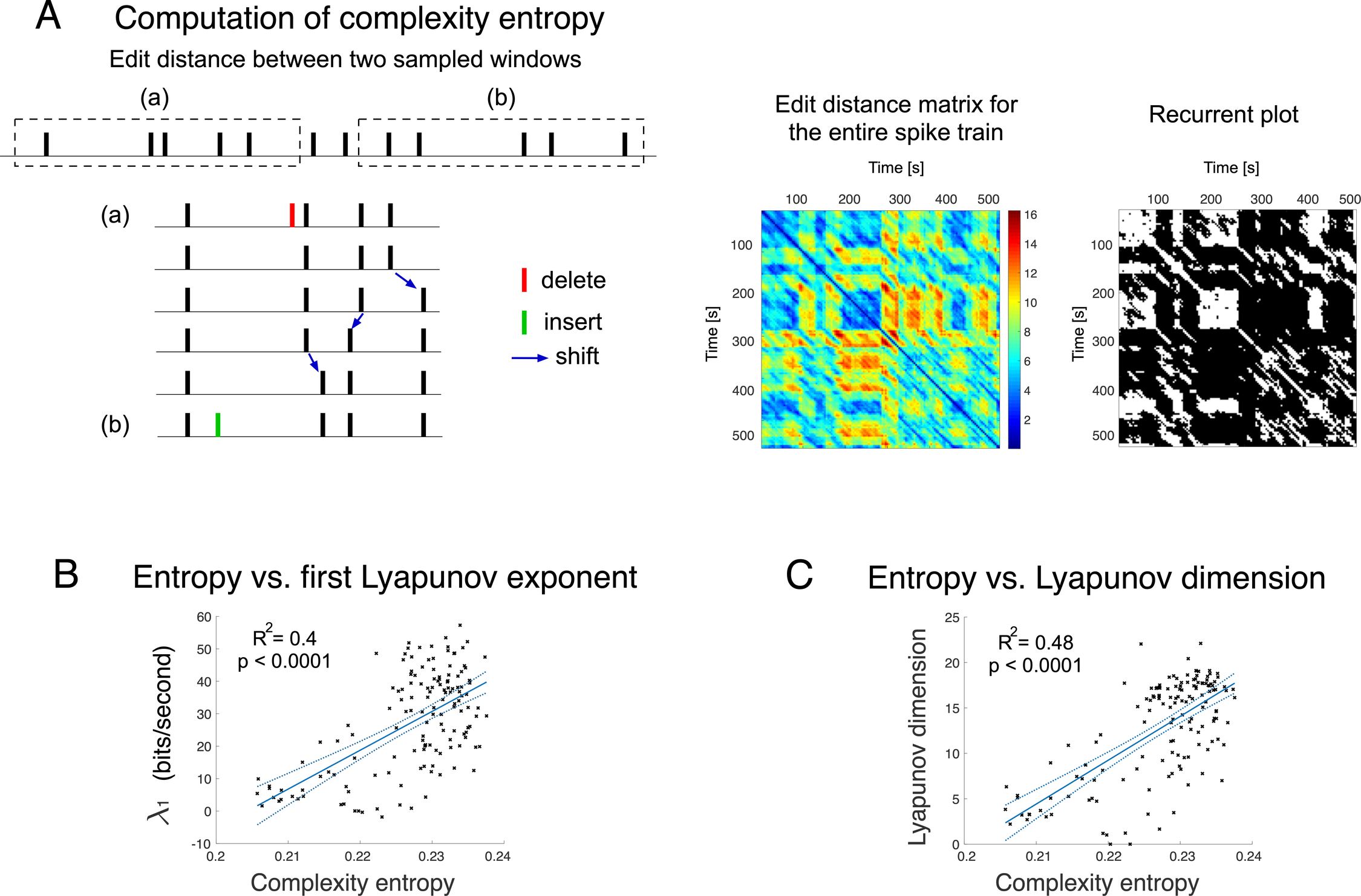

Supplement: S8 Fig — A: Illustration of edit distance computation between two sampled spike windows shows a sequence of elementary steps that convert the spike window (a) into (b). Each bar represents one spike. Allowed operations include deletion of a spike (shown in red), insertion of a spike (shown in green), or shifting a spike in time (blue arrows). Computation of edit distance for continuous sampling windows for the entire spike train constitutes the edit distance matrix. Then, the recurrent plot is constructed by binarizing the edit distance matrix. The points, whose values are smaller than the threshold, were plotted as white dots, otherwise as black dots. Complexity entropy is computed as the inverse of Shannon entropy, in terms of frequency distribution of the length of the diagonal lines of white dots [69]. B–C: Complexity entropy measured for a total of a hundred of parameter values (black crosses) in noise-free simulations showed strong positive correlations with the largest Lyapunov exponent λ1 (regression model: λ1 ~ 1 + entropy, R2 = 0.4, F-test: p < 0.0001, S8B Fig) and the Lyapunov dimension DKY (DKY ~ 1 + entropy, R2 = 0.48, F-test: p < 0.0001, S8C Fig). Solid cyan lines represent the fit of linear models with 95% CIs (dashed cyan lines). (TIF) [file pcbi.1008075.s008.tif]

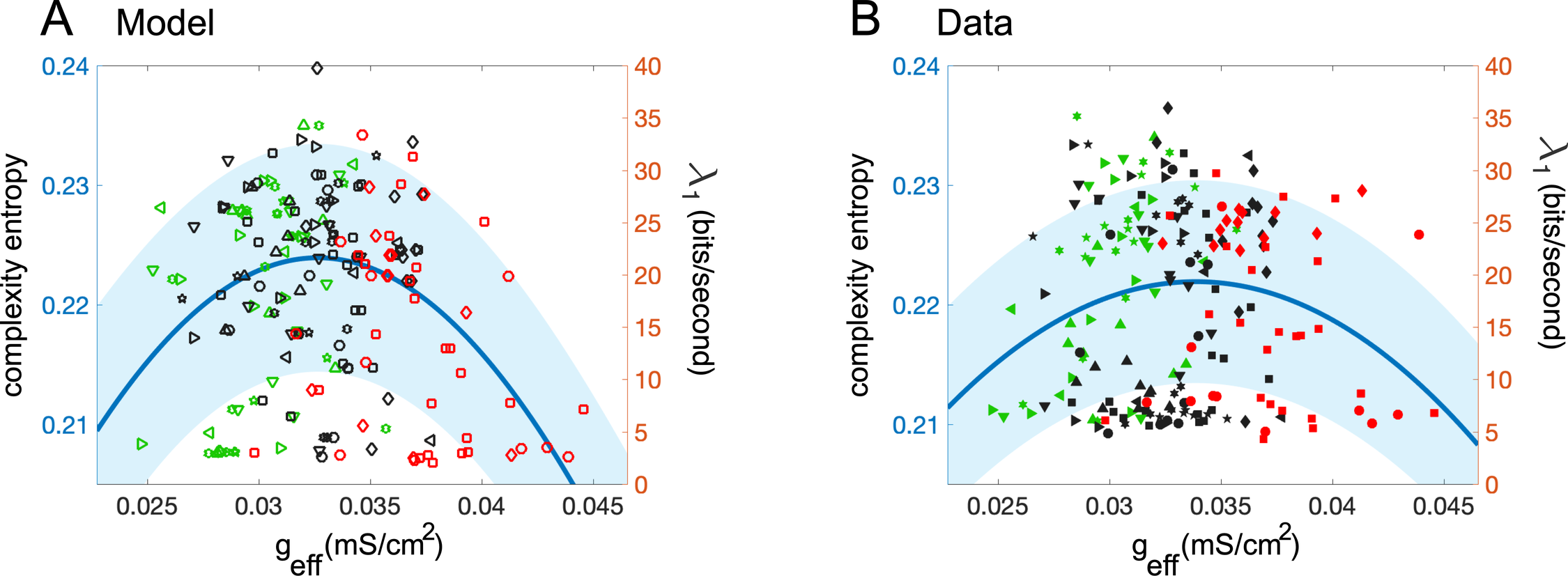

Supplement: S9 Fig — We investigated whether intermediate couplings maximize the complexity entropy by applying a non-parametric Gaussian Process regression model, which does not assume an explicit relationship between the coupling and the complexity entropy. Still, we observed inverted U-shaped curves maximized at around geff = 0.033 mS/cm2 for both the model (A) and the data (B). In sum, these results support the inverted U-shaped relationship between the effective coupling and complexity entropy. The right ordinates of A–B represent the first Lyapunov exponents approximated from the simulation data (see S8B Fig), indicating that intermediate couplings induce chaos. The shaded regions are of ±sem. (TIF) [file pcbi.1008075.s009.tif]

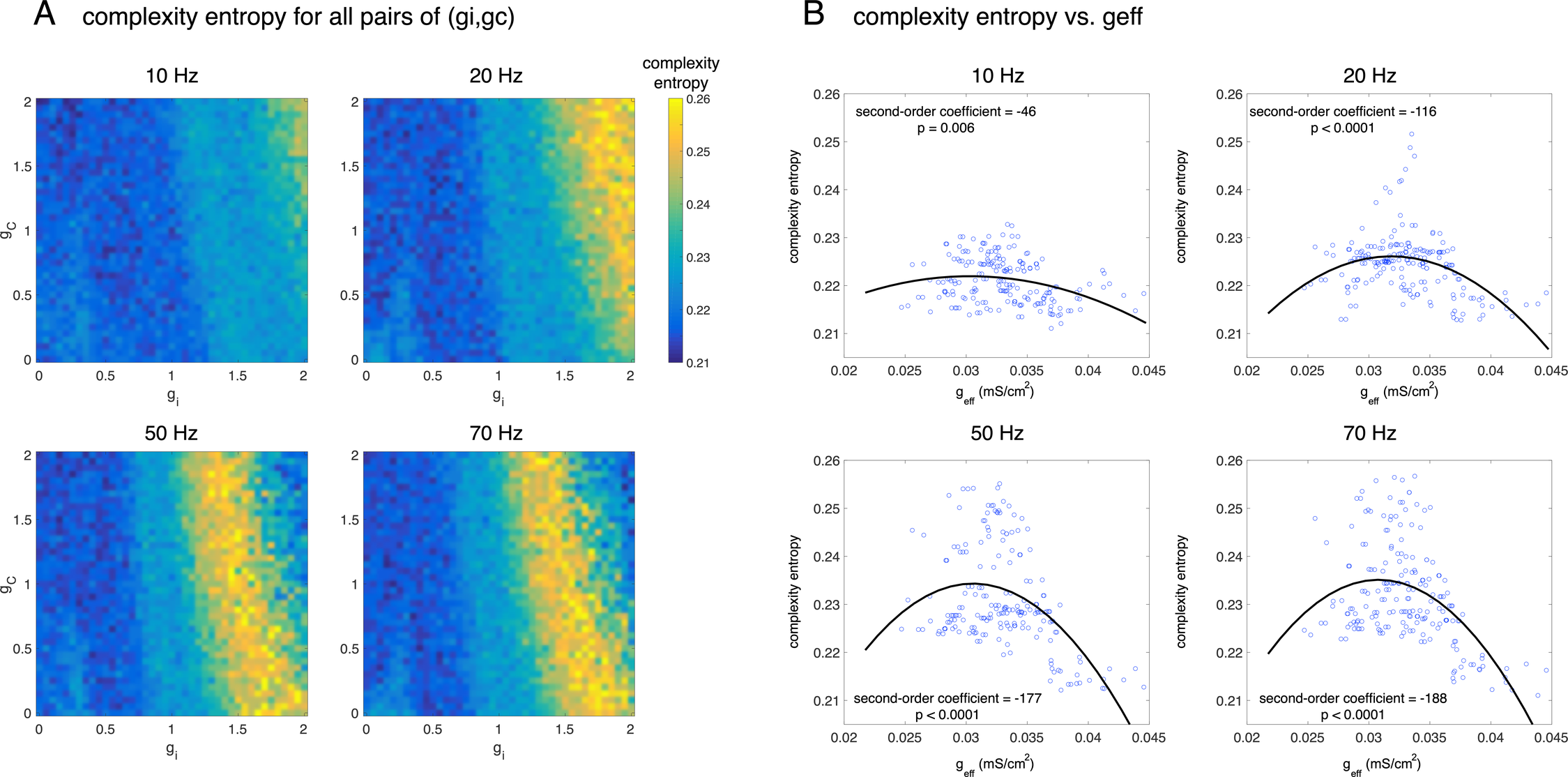

Supplement: S10 Fig — A: the pseudo-color heatmap of the complexity entropies, averaged across 9 model neurons, for all pairs of (gi, gc) in the range of 0–2 mS/cm2. The frequency of inhibitory synaptic input noise was varied in the range of 10–70 Hz. B: complexity entropy vs. effective coupling geff. In each model, a second-order regression (entropy ~ 1 + geff + geff2) fit was shown by black thick line. The second-order coefficient was negative and significant for all the models indicating the robustness of the inverted-U curve. (TIF) [file pcbi.1008075.s010.tif]
