# Supplementary material for: Electrical coupling controls dimensionality and chaotic firing of inferior olive neurons
Source: PLoS Comput Biol. 2020 Jul 30;16(7):e1008075. doi: 10.1371/journal.pcbi.1008075 (PMC7419012; doi:10.1371/journal.pcbi.1008075)
Supplement: Attachment — Submitted filename: IO_Response_to_Reviewers.docx [file pcbi.1008075.s011.docx]

*Dear Editor,*

*We are grateful to you for your kind editorial effort and to the three reviewers for their careful reading and constructive comments on the manuscript. We revised the paper according to the review comments and hope that it is now suitable for publication. Since there are many revised parts throughout the original manuscript, we provided two versions of the new manuscript: one with changes tracked and the other is clean without any markups. Please find below our detailed responses to each comment.*

*Sincerely yours,*

*Huu Hoang, Eric J. Lang, Yoshito Hirata, Isao T. Tokuda, Kazuyuki Aihara, Keisuke Toyama, Mitsuo Kawato and Nicolas Schweighofer*

===

**Editor**: Please make sure to address the concerns of all three reviewers. In addition, I would like you to add at least in the discussion your view on the impact of dimensionality in olivary activity on the induction of LTD (by increase in CF activity) as well as that of LTP (by a decrease in CF activity) in the context of learning (see also the comments from reviewer 3).

We thank the editor for handling our manuscript very nicely and giving this important advice. We first note that the dimensionality is relevant to signal representation across a neuronal population (in this case inferior olive cells) rather than in changes in the overall activity (i.e., average firing rate) of that population. That is, the dimensionality of olivary activity does not necessarily change when firing rates change; however, IO dimensionality may have an impact on the magnitude of any LTD/LTP induced by changes in activity levels and thereby accelerate learning. Specifically, early in learning, since the error magnitude is large, strong synchronization of active IO neurons (i.e low dimensionality) may trigger widespread and large changes in synaptic strength (LTP or LTD) in parallel fiber-Purkinje cell synapses among large numbers of Purkinje cells, resulting in fast learning. Conversely, late in learning, since the error magnitude becomes small, de-synchronized IO activity (i.e., high dimensionality) may optimally select the sites at which LTD/LTP occurs, resulting in sophisticated learning for fine tuning. We discuss these points in the discussion of the revised manuscript – Lines 528-540.

**Reviewer #1**: It is an interesting study that provides new insights about how olivo-cerebellar loop processes information and how the timing and learning hypothesis of cerebellar function can be reconciled. However, I have some points that I would like you to address:

1) During the whole study (Introduction, results and discussion), it was not mentioned at all the contribution of excitatory synapses to the modulation of coupling strength in the IO (Turececk et al 2014, Mathy et al 2014). For example, I saw you mentioned the study of Turececk et al, 2014. However, you seem to bypass the main findings of that paper which is that the activation of NMDA receptors enhances electrical coupling in weakly coupled neurons in the IO. Taking those results into account, you could also say that the observed enhancement of electrical coupling during the application of PIX in rats is due to an enhancement of excitation rather than absence of inhibition. I think both introduction and discussion sections should be rephrased in such a way that also should be considered the possibility of the effect of excitatory synapses on coupling strength in the IO. This is very crucial to keep in mind because it is known the close proximity of excitatory and inhibitory terminals to the electrically coupled dendritic spines in the glomeruli (De Zeeuw, 1990).

We thank the reviewer for pointing out this important point. We agree with the reviewer that excitatory synapses also play a role in controlling IO coupling strength. However, the effect of excitatory synapses is likely to be weaker than that of GABAergic synapses. In particular, it was recently shown that NMDAR-induced strengthening of IO coupling is restricted to neurons with nonadjacent somata that are weakly coupled, and that NMDAR activation does not enhance the maximum gain of coupling strength between directly adjacent neurons (Turececk et al. 2014). These results suggest that NMDAR activation via excitatory synapses plays a secondary role, one that is mainly to extend the coupled IO network. We discuss this possibility in both the introduction and discussion of the revised manuscript, citing Turececk et al. 2014 and Mathy et al., 2014. Please see Lines 94-98 and 477-499.

2) Figure 1, panel A: Do you think that the possible significant difference found between CBX and control conditions can be due to the possible direct actions of carbenoxolone on GABAa receptors (Tovar et al, 2009. Journal of Neurophysiology).

We thank the reviewer for indicating to us this interesting study showing possible effects of CBX on GABAa receptors. We performed a new analysis (see Figure below), which showed that CBX significantly reduces the inhibitory conductance *g_i_* from its control level (CON-CBX) as well as reducing *g_c_* (which was already described in the paper). We also note that when both experimental control groups are combined a significant drop in *g_i_* is still found with CBX (Figure 1A). This finding fits with the results of Tovar et al (2009) and is now discussed in the revised paper with (Tovar et al. 2009) being cited. See Lines 190-212.

*Figure: inhibitory conductance estimation for the individual conditions*

3) Figure 1, panel B: How can you explain the non-significant difference found in the gap junctional conductance between the control and PIX group?

This result agrees with physiological effect of PIX, which is known to have only effects on GABAergic conductance *g_i_* and not the gap-junctional conductance *g_c_*. Thus, *g_c_* remains unchanged between PIX and the control. However, the effective coupling *g_eff_* (computed from *g_i_* and *g_c_* by the Equation 1) was significantly reduced in the PIX compared with the CON condition (cf. Figure 1C).

**Reviewer #2**: The inferior olive is crucial for setting the activity level of the cerebellum, affecting (motor) execution as well as (motor) learning. The authors hypothesize that adjusting the coupling strength between inferior olivary neurons changes the dimensionality of inferior olivary output. The authors integrate (previous) experimental data with computational modeling to demonstrate that their ideas are biologically plausible.

Compared to other brain regions, neurons of the inferior olive (IO) have extraordinarily strong gap junctional coupling. This strong coupling has long been considered to be the basis of synchronization of IO output, and thus of complex spike firing by the postsynaptic Purkinje cells. However, it is now clear that the impact of coupling depends on the behavioral state of the animal and is strongly affected by anesthesia (see e.g. Mukamel et al., Neuron 2009). In awake animals, synaptic input to the IO strongly limits the impact of gap junctional coupling within the IO (see e.g. Negrello et al., PLoS Comp Biol 2019). Indeed, the authors noticed this impact of synaptic input (lines 159-160), but largely ignored this observation during their study.

The reviewer's caution about extrapolating from anesthetized (or any) preparation is well taken. The Mukamel paper (2009) compared complex spike synchrony in awake versus isoflurane anesthetized animals. They reported that synchrony was greatly reduced under anesthesia compared to the awake animal. However, while this may be true for isoflurane, the recordings in the present paper were made under ketamine/xylazine (K/X) anesthesia. One paper showed that synchrony patterns in awake and K/X animals are very similar (Lang 1999; J Neuroscience). Also, a second Ca- imaging study (Ozden 2009, J Neurosci) reported somewhat higher synchrony levels in awake animals compared to those found in our previous multielectrode paper; however, the reported difference in synchrony level is readily ascribable to methodological/definitional differences between that study and multielectrode recordings in anesthetized animals. For example, synchrony was defined using larger time bins in the imaging study, which would naturally lead to higher values. Further differences are detailed in a letter to the editor (see eLetter Lang 2009 that is attached to the main Ozden article). Basically, the letter shows that by adopting the same definitions of synchrony, and looking at equivalent cell pairs, essentially equal levels of synchrony are found under ketamine and in the awake animal. We discussed this point in the revised manuscript (Lines 394-416), and we included (Mukamel et al. 2009; Neegrello et al. 2019).

However, the reviewer is correct to point out that, at the network level, synaptic input strongly affects the oscillatory activity and thus may have a large impact on the coupling. We in fact considered this impact by estimating the coupling in the four models of a varied synaptic input strength, i.e. the inhibitory synaptic input frequency was varied 10-70 Hz as observed in the slice preparation (Najac and Raman, 2015). The coupling estimates were varied across the four models but such variance was averaged by a model-averaging approach to obtain the final estimates of the coupling (c.f Figure S4C).

While their idea – controlling the dimensionality by adjusting coupling strength within the IO – is interesting and appealing, the use of experimental data from anesthetized animals restricts the interpretational value of this study. Several studies, using predominantly optical recording techniques, but there are some electrophysiological studies as well, have published complex spike recordings of multiple Purkinje cells in awake animals and related to specific tasks. Relating the ideas of the current manuscript to (at least) one of the awake datasets would strengthen the study.

We thank the reviewer for this reasonable suggestion. As described in the previous response, synchrony levels and firing rates of the olivocerebellar system are similar in awake and K/X anesthetized animals, suggesting that the system is operating in a similar part of the state space and so the findings of the current paper are relevant to the awake state (we noted this in the discussion – Line 394-416). While recordings in awake animals and analyzing the effect of change in IO coupling would definitely be an interesting and important study, it is very challenging to distinguish the synchrony effects of coupling strength and synaptic input in awake animals because it is much harder to manipulate each of these factors independently and thus have two conditions in the same animal for comparison. While we could compare different groups of animals, inter-animal variability could become an issue and would require large numbers of animals. We thus believe that analysis of IO data from awake animals is out of the scope of our (already quite long) study and best left for future work.

In Fig. 3, the authors suggest – but do not show directly – that synchrony is linearly related to dimensionality. This seems an intuitive, if not trivial, result. It would help to show whether changes in synchrony that are physiologically relevant are indeed coupled to changes in dimensionality. This would demonstrate whether IO synchrony is indeed modulated during different phases of motor adaptation and learning, as suggested by the last paragraph of the Discussion.

We thank the reviewer for this important suggestion. For precisely showing changes in synchrony are coupled to changes in dimensionality, the timescales for computing those two quantities must be correlated. However, in the original manuscript, we used the timescale of 1 ms for synchrony as in the electrophysiological studies (Lang 1996, Lang et al. 2002) but that of dimensionality computation (i.e the interval of sampling) was 2 seconds. As a result, we could not observe a clear relationship between synchrony change vs. dimensionality change in individual animals.

We therefore have performed a revised analysis with the two identical timescales of 10 msec and confirmed that the change in synchrony is linearly related with the change in dimensionality for individual animals, i.e. an increase in synchrony leads to a decrease in dimensionality.

Regarding the physiologically meaningful definition of the synchrony of Purkinje complex spike, time bins of 1-10 ms were commonly used in literature (Sasaki et al., 1989; Long et al. 2002). This value was derived empirically using the shape of a typical cross-correlogram between two highly correlated Purkinje cells, where the central peak has a width between 1-10 ms. A second line of reasoning is based on the gap junction properties themselves. Recordings from (Llinas et al., 1974) show that activation of one IO cell leads to a depolarizing potential in coupled cells on a very short, ms time scale, consistent with the peaks of complex spike cross-correlograms. In a more recent paper (Tang et al., 2019, elife), the authors first showed that synchronous complex spike activity, defined traditionally with a short ~5 ms window, had a major inhibitory effect on nuclear activity. They then relaxed the time window to see how this effect dissipated. In most cases, the inhibitory effect weakened dramatically when the window exceeded 10 ms. In sum, these studies taken together suggest that the gap junction mechanism should generate synchrony on a timescale ≤10 ms. In the revised manuscript, we used the time bins of 10 ms for computing the synchrony as well as the dimensionality. Note, however, that this relationship observed in the actual IO data does not depend crucially on the exact choice of time bin, as shown in the figure below in which we used 1, 5, and 10 ms time bins.

*Figure: change of synchrony vs. change of dimensionality in the 9 animals (6 CBX – green symbols and 3 PIX – red symbols) by the drug condition and its control with the three different timescales (1, 5 and 10 ms).*

Furthermore, we conducted new simulations of complex spike activity to confirm that the synchrony related to the dimensionality. Briefly, we independently generated Poisson spike trains with the firing rate of 1Hz for a total of n=20 neurons. To manipulate the correlation of the spike trains, the parameter p (ranged in 0-1) was defined as a portion of the random spike events of a reference neuron (neuron #1) to be “copied” to the other neurons (#2-20). For each spike event copied, another random spike event was removed from the spike train being manipulated (neuron #2-20). If p=0, the spike trains are totally independent. If p=1, all spike events of neuron #1 are copied to the other neurons #2-20, resulting in complete synchrony of the spike trains (Staude et al. 2010). For the generated spike trains, as the parameter p varies (5 trials each), we computed the synchrony and the dimensionality, both with 10 ms time bins, and found a robust approximately linear relationship between these two measures. These results thus strongly support our hypothesis that synchrony is a biologically plausible mechanism to control the dimensionality of neural population. We included this simulation analysis as the figure S7 in the revised manuscript.

*Figure: Simulations of the correlated Poisson spike trains show strong relationship between synchrony and the dimensionality.*

The authors present direct comparisons of biological data and modeling data (e.g., Fig. 2). This comparison would benefit from providing more details (also in the Figure; not only summarized in the text). While the impact of the drugs on firing rate is obvious, more relevant measures like synchrony are not illustrated in a direct comparison.

Thank you very much for your advice. In the revised manuscript, we have added more details in direct comparisons of biological data and modeling data, both in Figure 2 and in the text (Lines 222-234), as suggested by the reviewer.

In short, the authors present an interesting hypothesis: that the degree of gap junctional coupling controls the dimensionality / entropy of the IO output via modulation of IO synchrony. Their data suggest that the amplitude of changes in gap junctional coupling required for having an impact on dimensionality / entropy are realistic given experimental data. However, the experimental data are recorded under anesthesia, which is known to affect IO synchrony, and variations in coupling are only linked to pharmacological intervention and not to behaviorally relevant changes, clearly limiting the relevance of this study in its current form.

**Reviewer #3**: I apologize in advance if the review sounds harsh.

The paper puts forward a story about the putative functional consequences of an adaptive modulation of gap junctional plasticity in the inferior olive in the course of learning. It extends a well-validated model of inferior olivary cells to a network in order to study the effect of gap junctional modulation on ‘dimensionality’ of the system.

I have multiple problems with the paper in its current form, and I am afraid I cannot recommend publication in PLoS Comp. Bio. The argumentation in the paper is rather shallow and repetitive, mostly recapitulating earlier arguments but with scant new evidence.

The whole introduction reads as a discussion of the functional consequences of an assumption. It heavily depends on a particular theoretical framework, that which upholsters the view that cerebellar function is to perform a basic form of supervised learning. It uses the word dimensionality profusely, but confusingly. It identifies reduction of dimensionality in the control problem with the reduction of dimensionality in synchronizing coupled oscillators, though the logic for this step are not spelled out.

We thank the reviewer for carefully reading our manuscript and giving us many constructive comments. In the revised manuscript, we thoroughly revise the manuscript, and notably the introduction and the discussion, to emphasize functional consequence of dimensionality/chaos control in motor control as well as to avoid confusions.

Furthermore, the authors argue here (and previously) that a reduction of dimensionality is a desirable end state for motor control, but that is not at all a given, and depends very much on the type of control problem (for example, smooth grasp requires higher correlation between output control variables).

The reviewer may have misunderstood the point we were trying to make, probably because the text was not sufficiently clear. We did not argue that “a reduction of dimensionality is a desirable end state of motor control”. Instead, we argued that dimensionality should be dynamically controlled depending upon the stage of learning or control problem. For motor learning, the dimensionality at an early stage should be small due to a lack of training samples and is eventually increased as the learning proceeds. For motor control, high dimensional signals would indeed be used for the fine grain motor control commands that are needed for precise motor coordination (Hoogland et al, 2015). In any case, synchronization via coupling is one of the feasible mechanisms for controlling the dimensionality required for efficient motor learning/control. We have edited the end of the discussion to try to better convey this message. In addition, we hope that our largely revised introduction, which places less emphasis on motor learning, will address the reviewer’s concern.

Major points:

1. Heavy usage of abstract terms with vague and weak links to actual problems of motor control:

- The use of the word ‘dimension’ is too loose. There seems to be a confused use of the term ‘reduction of dimensionality’, where the authors seem to assume that the reduction of dimensionality of an oscillatory system due to synchrony is akin to what happens in neural networks (such as in auto-encoders). This identification is highly problematic, because it is not clear which are the variables being encoded/decoded.

In addition of mathematically defined the “dimensionality” of the neural state in Equation (5), we now clearly define it in the introduction (Lines 101-102).

Even though the ultimate goal of dimension reduction is the same, our proposal of dimension reduction of an oscillatory system via coupling-induced synchronization contrasts other neural networks (e.g auto-encoders) in that we propose a framework of neural communication among the neurons for transmitting information rather than approximating a function that maps the data from high-dimensional space to low-dimensional space (i.e, encoding/decoding). In most artificial neural networks, such encoding/decoding scheme creates a black box on their mechanisms in reducing the dimension in the data. For instance, there is no meaningful link between the weights and the function being approximated, or which variables in the data are irrelevant is an open problem. In contrast, the core idea of our proposal is that coupling provides a biologically plausible mechanism to achieve stable and reliable transitions between different oscillatory regimes depending upon the required task. Strong couplings induce robust phase-locked oscillation in coupled neurons: the signal to N neurons can be conceptually transmitted by M clusters of phase-locked neurons (M<N) (Williamson et al. 2016). Thus, the dimensionality of N neurons reduces to M. Conversely, intermediate/weak couplings de-synchronize the neurons. In such cases, each neuron may transmit different aspects of the signal at its own rhythm, thus the dimensionality of the network is maximized. It is worth noting that the coupling strength discussed above is relative to the input signal since both factors may have an interplay effect on oscillatory dynamics of the neurons. We have added some text in the discussion (see Lines 433-444) comparing dimensionality reduction in networks like auto-encoders to our proposed scheme.

- Substantial differences are expected in coupling conductances in real networks. Small perturbations would be sufficient to create mounting phase disparities in the cell populations, unless these perturbations are correlated.

We entirely agree with the reviewer regarding this point. In our model of 3x3 IO neurons, each neuron is connected to its four neighbors by the gap-junctional conductance (*g_c_*), whose strength was drawn from a uniform distribution with the maximum deviation set at ± 20% of the mean (Lines 603-604). Thus, the heterogeneity of coupling in our model network reflects substantial differences in coupling conductances in real networks (Hoge et al. 2011).

- In an oscillatory system driven by noise, the use of chaos as a functional property is contentious. The time scales where sensitivity to initial conditions would have a measurable effect are long with respect to motor behavior. More plausible sources of phase variability exist which are ‘transient’, rather than asymptotic, as required by the use of the term ‘chaos’.

Here again, we agree with the reviewer regarding this point. Noise may indeed provide an alternative source for irregularity and phase variability. There exist numerous studies on utilizing noise in neuronal modeling, e.g., synaptic noise in cortical networks (Destexhe and Contreras, 2006). However, the concept of noise creates a black box on their generation mechanism and the source of noise in neuronal activity is largely unclear. In the studies of cortical networks, on the other hand, it has been shown that a network of lognormally-distributed IPSPs can generate spontaneous firing activities, where deterministic chaos plays the role of noise (Teramae et al., 2012; Ikegaya et al., 2013; Kriener et al., 2014). These studies suggest that deterministic chaos, which is induced by intrinsic properties of the neural network (e.g. heterogeneous synaptic connections or coupling strengths), and noise may potentially co-exist and complement each other (Kada et al. 2018).

The issue of time scale is very important. The phase variability induced by chaos can be only within a time scale of the IO firing frequencies, which is very low and thus might be only useful for efficient motor learning. In particular, we have shown, by simulations, that in the chaotic regime of IO network the increase in information transmission in IO neurons is achieved via distributing-frequency components of the error inputs over irregular and non-phase-locked spikes (Schweighofer et al., 2004). Desynchronization is indeed necessary for scattering the spike timings of each neuron to increase the time resolution of the population rate coding. Then, the complete continuous error signal can be reconstructed by spatial integration across Purkinje cells within a micro-complex and via temporal integration for each Purkinje cell via cumulative effects of LTD. In contrast, for online motor control, IO neurons could significantly contribute to motor commands primarily when it is operating in a relatively synchronized state. This is because when complex spike activity is desynchronized, it may be less distinguishable from simple spike activity.

- Chaos in a slowly oscillating system would only be measurable in longer time scales (>>100ms) . But in long time scales the sensitive dependence of initial conditions will likely be swamped under the effects of (1) input noise (2) intrinsic plasticity (3) short term synaptic plasticity (4) changes of rectifying properties in gap junctions. In the absence of parameter space analysis

We agree and thank the reviewer for this excellent comment. To address this point, we conducted a new parameter analysis of the complexity entropy (our measure of chaotic behavior) using the IO model. In particular, we examined the change of complexity entropies while varying the three parameters: (1) frequency of inhibitory synaptic input noise (10-70 Hz), and (2) the inhibitory conductance *g_i_*, and (3) the gap-junctional conductance *g_c_*.

As shown in the figure below (also included in new supplementary figure S10A), complexity entropies were slightly shifted to the lower *g_i_* values when increasing the frequency of inhibitory synaptic input noise. This is probably because strong synaptic inhibitory inputs suppress the neuron activity and thus reduce the complexity entropies.

*Figure: Effects of the intrinsic properties of the network model on complexity entropies. The pseudo-color heatmap of the complexity entropies, averaged across 9 model neurons, for all pairs of (g_i_, g_c_) in the range of [0, 2] mS/cm^2^ in the model. The frequency of inhibitory synaptic input noise was varied in the range of 10-70 Hz.*

In addition, and importantly, significant inverted-U shape, maximized at around *g_eff_* = 0.032 mS/cm^2^, was still observed in the individual models for the range of *g_eff_* estimated in the data for all noise levels, as shown in the figure below (now new supplementary figure S10B).

*Figure: complexity entropy vs. estimated effective coupling g_eff_. In each model, a second-order regression (entropy ~ 1 + g_eff_ + g_eff_^2^) fit was shown by black thick line. Also, the second-order coefficient and significance level were displayed.*

In sum, this new parameter analysis shows that whereas the firing frequency of synaptic input changes the landscape of the complexity entropy, a measure of chaos, our main conclusion does not change. Thus, the inverted-U shapes observed in individual models as well as in the model-averaging approach reported in the text (Figure 4A) supported our hypothesis that intermediate effective couplings maximize the complexity entropies.

- Many processes influence and drive synchrony, most importantly, input correlations. The level of synchrony induced by coupling is likely smaller than the correlations from GABAergic and Glutamatergic sources.

The reviewer raises an important point. The issue of how correlated synaptic input affects the synchrony patterns has been studied experimentally (Lang, 2001 JNeurosci; Lang 2002, JNeurophysiol; Lang et al 1996 JNeurophysiol). The evidence indicates that the patterns of synchrony strongly reflect the coupling of IO neurons under a range of conditions, though of course the correlations in afferents can play a role as well.

Regarding inhibitory inputs, it is important to point out that the highest levels of synchrony occur when GABAergic input to the IO is blocked or destroyed (Lang et al. 1996). These levels are high compared to those for spontaneous activity in either the spontaneous or awake state. So, if anything inhibitory synaptic input lowers synchrony rather than being the prime cause of it. In addition, the GABAergic synapses in the IO appear to function mainly through asynchronous release of GABA (Best and Regehr, 2009, Neuron) that reflects average release over relatively long time scales (10's to 100's of ms). This would seem to preclude the transformation of the precise timing of GABAergic afferents into IO synchrony patterns. Furthermore, the cerebellar nuclear cells that project to the IO have synaptic and electrophysiological properties indicating that they function as neuronal integrators over periods of 10's of milliseconds (see papers by Indira Raman), so it is unlikely that they would show precisely correlated activity amongst themselves.

The specific effect of synchrony in excitatory inputs was investigated with stimulation of IO afferents from the motor cortex. Such stimulation increased overall synchrony levels, but the spatial distribution of synchronous activity remained very similar to that of spontaneous activity (Lang 2002). This argues for the coupling among IO neurons to be a major determinant of the synchrony patterns, as the motor cortex stimuli produced afferent patterns that almost certainly are very different from spontaneous patterns, and yet, the synchrony patterns were very similar. Moreover, the spatial distribution (i.e., highest synchrony among Purkinje cells in the same rostrocaudal strip of cortex) seen in these cases is essentially the same as is present after blocking excitatory input to the IO (Lang 2001) and thus cannot be dependent correlations in excitatory input. A further argument for the importance of coupling is that the synchrony patterns are very similar in ketamine anesthetized and awake animals (Lang 1999 JNeurosci), because the cerebral cortical patterns (and hence descending excitatory drive to the IO) are very different in these two cases and yet the synchrony patterns are similar. In sum, the basic pattern of synchrony seems to be strongly determined by the coupling pattern within the IO, even when faced with very large changes in the correlation of excitatory input patterns.

Finally, when coupling in the IO is blocked with carbenoxolone or absent (connexin36 knockout mouse) there is little to no synchrony in complex spike activity, despite the presence of afferent input. So, baseline synchrony levels are entirely dependent on IO coupling (Blenkinsop and Lang 2006 JNeurosci; Marshall et al., 2007 Cerebellum).

In sum, the above results suggest that coupling contributes significantly to the synchrony patterns normally observed. We have added the following text in the discussion (Lines 477-499) to address this point.

- “Low dimension components of error signal” Usually the error signal is assumed to be a coarse representation of an organismic-level event.

In the original manuscript, by “low-dimensional components” we meant low-frequency components of the error signal, which may be considered as a low-pass filtered signal of the complex actual error input. Despite the low temporal resolution, the low-frequency component of the error may include essential information to correct gross features of movements. In the revised manuscript, we used the term “low-frequency components” to avoid misunderstanding (see Line 513).

- Similarly, it is not always clear what the DoF that the paper is referring to. Motor synergies highly constrain the number of DoF in motor output systems. Only twitches and the like fit into ‘reduction of DoF’.

We removed the term DoF and related discussion in the revised manuscript. We agreed to that this could lead to misunderstanding and was not crucial for our argument.

2. Cerebellum as supervised learning machine: tacit acceptance of the theory that supervised learning in the cerebellum is driven primarily by the CF related LTD.

- The idea is that reduction of coupling conductance and synchrony co-occurs with training in the system presumes that the main learning mechanism in the cerebellum is CS driven PF plasticity, i.e., supervised learning. Though this is a common belief, there are continued and substantial problems with the idea. Particularly as one attempts to create more complex control systems with more degrees of freedom in 3D space, the requirements for a motor error signal rise substantially. Though this is not the place for a full discussion of the problems with this assumption, given the number of years that the hypothesis had to prove its ability to explain high level control and acquisition, a more careful presentation of the theory is called for.

We agree with the reviewer that the original manuscript heavily relied on the theory of supervised learning in the cerebellum. To address these concerns, in the revised manuscript, we largely rewrote the introduction and discussion.

- The paper uses the literature to justify the assumption that GABAergic sources reduce coupling between cells. In the paper mentioned, they have not controlled for the effects of inhibition in the measured reduction of coupling conductance. It remains highly plausible that reduction of coupling is simply a consequence of shunting of oscillations rather than a direct effect on the coupling values. Hence, adaptation of coupling is not induced by GABAergic sources, but simply a momentary reduction of oscillatory amplitude.

We understand the reviewer’s concern that GABAergic sources may also have an inhibitory effect on IO oscillation (Bazzigaluppi et al., 2012). In a general context, the change in complex spike activity probably reflects changes in coupling strength and excitability of IO neurons, both of which are potentially affected by GABAergic sources. To examine this possibility, we simulated the four models varying different levels of inhibitory drive (i.e. firing frequency of inhibitory synaptic inputs). Such inhibitory drive changes the overall excitability of the IO model neurons without changing their couplings. The effective coupling strengths estimated from the IO spiking data was slightly varied across the models (see figure below, now added as the supplemental figure S4C in the main text), indicating the possible effect of GABAergic sources on excitability of IO neurons. However, a same tendency in the change of effective coupling strengths in the three conditions (i.e., smaller values in the CBX condition and larger values in the PIX condition compared to the CON) was observed across the models, supporting direct controls of GABAergic sources on the coupling strengths. Thus, in our study, we marginalized out the effect of GABAergic sources on excitability of IO neurons by the model-averaging approach (cf. Methods for details). We made a remark to discuss this possibility in the revised manuscript – Lines 483-492.

*Figure: the estimated effective coupling, g_eff_, in the three data conditions for varying levels of inhibitory synaptic inputs.*

**References**

Bazzigaluppi, P., Ruigrok, T., Saisan, P., de Zeeuw, C.I., and de Jeu, M. (2012). Properties of the Nucleo-Olivary Pathway: An In Vivo Whole-Cell Patch Clamp Study. PLoS One 7.

Best, A.R., and Regehr, W.G. (2009). Inhibitory Regulation of Electrically Coupled Neurons in the Inferior Olive Is Mediated by Asynchronous Release of GABA. Neuron *62*, 555–565.

Blenkinsop, T.A., and Lang, E.J. (2006). Block of Inferior Olive Gap Junctional Coupling Decreases Purkinje Cell Complex Spike Synchrony and Rhythmicity. J. Neurosci. *26*, 1739–1748.

Destexhe A. and Contreras D. (2006) Neuronal computations with stochastic network states. Science. 314(5796):85-90.

Hoge, G.J., Davidson, K.G., Yasumura, T., Castillo, P.E., Rash, J.E., and Pereda, A.E. (2011). The extent and strength of electrical coupling between inferior olivary neurons is heterogeneous. J. Neurophysiol. *105*, 1089–1101.

Hoogland, T.M., De Gruijl, J.R., Witter, L., Canto, C.B., De Zeeuw, C.I. (2015). Role of synchronous activation of cerebellar Purkinje cell ensembles in multi-joint movement control. Curr Biol. 25:1157–65.

Ikegaya, Y., Sasaki, T., Ishikawa, D., Honma, N., Tao, K., Takahashi, N., et al. (2013). Interpyramid spike transmission stabilizes the sparseness of recurrent network activity. Cereb. Cortex 23, 293–304. doi: 10.1093/cercor/bhs006

Kada H, Teramae J and Tokuda IT (2018) Highly Heterogeneous Excitatory Connections Require Less Amount of Noise to Sustain Firing Activities in Cortical Networks. Front. Comput. Neurosci. 12:104. doi: 10.3389/fncom.2018.00104

Kriener, B., Enger, H., Tetzlaff, T., Plesser, H. E., Gewaltig, M.-O., and Einevoll, G. T. (2014). Dynamics of self-sustained asynchronous-irregular activity in random networks of spiking neurons with strong synapses. Front. Comput. Neurosci. 8:136. doi: 10.3389/fncom.2014.00136

Lang E.J. (2001) Organization of olivocerebellar activity in the absence of excitatory glutamatergic input. J Neurosci. 21:1663-75.

Lang, E.J. (2002). GABAergic and glutamatergic modulation of spontaneous and motor-cortex-evoked complex spike activity. J. Neurophysiol. *87*, 1993–2008.

Lang, E.J., Sugihara, I., and Llinás, R. (1996). GABAergic modulation of complex spike activity by the cerebellar nucleoolivary pathway in rat. J. Neurophysiol. *76*, 255–275.

Lang E.J., Sugihara I., Welsh J.P. and Llinás R. (1999). Patterns of Spontaneous Purkinje Cell Complex Spike Activity in the Awake Rat. J Neurosci. 19 (7) 2728-2739.

Llinás, R., Baker R., and Sotelo C. (1974). Electrotonic coupling between neurons in cat inferior olive. J. Neurophysiol. 37(3), 560-571.

Long M.A., Deans M.R., Paul D.L., Connors B.W. (2002) Rhythmicity without synchrony in the electrically uncoupled inferior olive. J Neurosci. 22(24):10898-905.

Marshall S.P., van der Giessen R.S., de Zeeuw C.I. and Lang E.J. (2007). Altered olivocerebellar activity patterns in the connexin36 knockout mouse. Cerebellum. 6(4):287-99.

Mukamel EA, Nimmerjahn A, Schnitzer MJ (2009). Automated analysis of cellular signals from large-scale calcium imaging data. Neuron. 63(6):747-60.

Najac, M., and Raman I. M. (2015). Integration of Purkinje Cell Inhibition by Cerebellar Nucleo-Olivary Neurons. J. Neurosci. *35(2)*, 544–549.

Ozden I., Sullivan M.R., Lee H.M. and Wang S.S.H. (2009). Reliable Coding Emerges from Coactivation of Climbing Fibers in Microbands of Cerebellar Purkinje Neurons. J. Neurosci. 29 (34) 10463-10473.

Sasaki K., Bower J.M., Llinás R. (1989). Multiple Purkinje Cell Recording in Rodent Cerebellar Cortex. Eur J Neurosci. 1(6):572-586.

Schweighofer, N., Doya, K., Fukai, H., Chiron, J.V., Furukawa, T., and Kawato, M. (2004). Chaos may enhance information transmission in the inferior olive. Proc. Natl. Acad. Sci. *101*, 4655–4660.

Staude B, Grün S and Rotter S (2010) Higher-order correlations in non-stationary parallel spike trains: statistical modeling and inference. Front. Comput. Neurosci. 4:16. doi: 10.3389/fncom.2010.00016.

Tang T., Blenkinsop T.A. and Lang E.J. (2019). Complex spike synchrony dependent modulation of rat deep cerebellar nuclear activity. eLife. 8:e40101. doi: 10.7554/eLife.40101.

Teramae, J.-N., Tsubo, Y., and Fukai, T. (2012). Optimal spike-based communication in excitable networks with strong-sparse and weak-dense links. Sci. Rep. 2:485. doi: 10.1038/srep00485

Tovar KR., Maher BJ. and Westbrook GL. (2009) Direct Actions of Carbenoxolone on Synaptic Transmission and Neuronal Membrane Properties, J Neurophysiol 102: 974–978.

Turecek, J., Yuen, G.S., Han, V.Z., Zeng, X.H., Bayer, K.U., and Welsh, J.P. (2014). NMDA receptor activation strengthens weak electrical coupling in mammalian brain. Neuron *81*, 1375–1388.

Williamson RC, Cowley BR, Litwin-Kumar A, Doiron B, Kohn A, Smith MA, et al. (2016) Scaling Properties of Dimensionality Reduction for Neural Populations and Network Models. PLoS Comput Biol 12(12): e1005141. https://doi.org/10.1371/journal.pcbi.1005141
