# Supplementary material for: Electrical coupling controls dimensionality and chaotic firing of inferior olive neurons
Source: PLoS Comput Biol. 2020 Jul 30;16(7):e1008075. doi: 10.1371/journal.pcbi.1008075 (PMC7419012; doi:10.1371/journal.pcbi.1008075)
Supplement: Attachment — Submitted filename: IO_2R_Reply.docx [file pcbi.1008075.s012.docx]

*Dear Editor,*

*We are grateful to you for your kind editorial effort and to the reviewers for their careful reading and supportive comments on the manuscript. We were glad that the two reviewers found that our manuscript has been substantially improved. We revised the paper according to the review comments and hope that it is now suitable for publication. Please find below our detailed responses to each comment. The revised parts are marked in red in the main text.*

*Sincerely yours,*

*Huu Hoang, Eric J. Lang, Yoshito Hirata, Isao T. Tokuda, Kazuyuki Aihara, Keisuke Toyama, Mitsuo Kawato and Nicolas Schweighofer*

---

**Reviewer #1:** This new version of the manuscript has been improved substantially. You have answered all my questions and include my suggestions. Moreover, you have clarified some important concepts such as dimensionality which provides the reader with a better understating of the main message of this study.

We are grateful to the supportive comments from the reviewer.

I only have minor remarks that I would like you to address:

Legend of Figure 2: Should the CBX animal show ‘chaotic’ firing instead of ‘repetitive’ whereas the control animal should exhibit ‘repetitive’ firing and not ‘chaotic?

We agree with the reviewer regarding this point. We confirmed by temporal features - including auto-correlations and local variation (LV) - that the control animals show more oscillatory and less irregular firings than the CBX animals. In the revised manuscript, we changed the legend of Figure 2 as “oscillatory spiking” for control animals and “irregular spiking” for CBX animals (Line 239-240).

Figure 3: Would you expect a different relationship between dimensionality and synchrony if you would extract the coefficient of variation of adjacent interspike intervals (CV2) instead of the average firing frequency in order to calculate the dimensionality of complex spike activity?

We thank the reviewer for suggesting an interesting idea. Essentially, dimensionality is a quantitative measure of the similarity in firing dynamics of the neurons in a neuronal population. Therefore, calculation of the dimensionality from different temporal features of firing dynamics may yield similar results.

We conducted a new analysis to test the idea suggested by the reviewer. In particular, we extracted the coefficient of variance of adjacent inter-spike intervals (CV2) from the spike train of each neuron as:

$$CV2=2\frac{\left| ISI_{k}-ISI_{k+1} \right|}{ISI_{k}+ISI_{k+1}}$$

The CV2 value computed from the two adjacent ISIs was allocated at the timing of the middle spike. As a result, we obtained a “raster” of CV2 values at different spike timings for each neuron (panel A in the below figure). Finally, the dimensionality was calculated by PCA from the sum of CV2 values in a moving window with the width of 20 seconds and the interval of 10 milli-second (i.e the timescale of synchrony). As expected, the relationships between the dimensionality and the synchrony as well as the effective coupling remain unchanged (panels B-D).

*Figure legend: A: CV2 values at spike timings of 4 neurons in a representative control animal (animal #1). B: dimensionality, computed from the sum of CV2 values in the window of 20 second, for the three data conditions. C: change in synchrony is coupled with change in the dimensionality (timescale of 10 ms). D: dimensionality as a function of effective coupling g_eff_ shows a negative correlation r = -0.4 (p = 0.1).*

Figure 3B: Can you explain why is there no significant difference between both the PIX and control group and CBX and control group?

The non-significant difference in dimensionality between both the PIX/CBX and control group was mainly due to 1) the small number of samples (6 animals in CBX-CON and 3 animals in PIX-CON experiments), and 2) the large variance in the dimensionality across the animals in the individual conditions. Taken together, we cannot observe a statistical difference between both the PIX and control group and CBX and control group, although the magnitude of difference in the dimensionality was large (approximately 2-folds) among the conditions. We briefly mentioned this point in the revised manuscript (Line 268-272)

Discussion: The last part of the discussion is redundant when it’s mentioned the role of synchrony at early and late stages of learning. It can be more concise.

We thank the reviewer for pointing out that redundancy in the discussion. We made it more concise in the revised manuscript (Line 515-537).

---

**Reviewer #2**: I would like to thank the Authors for taking the time to substantiate their manuscript.

Synchrony within the inferior olive, and thereby between complex spikes of downstream Purkinje cells, is an abundant phenomenon. In this paper, the authors argue that gap junctional coupling between IO cells can affect synchrony and that this in turn affects the dimensionality of the olivary-cerebellar system. Intuitively, there has to be a relation between synchrony and dimensionality: all cells firing in perfect synchrony minimize and all cells firing chaotically maximize entropy. In the first round of review, I mainly referred to the biological relevance of the current study. In response to my question regarding the relation between synchrony and dimensionality (which was suggested, but not shown), the authors now demonstrate this relation in the new Fig. S7.

I am afraid that my concerns were not fully addressed. I remarked that such a relation between synchrony and dimensionality is (almost) trivial. I indicated that it would help to provide evidence that the synchrony varies under physiologically relevant situations to such a degree that changes in dimensionality become apparent. Related to this issue, I have also encouraged the inclusion of awake data.

In response to the latter remark (on the inclusion of awake data) the authors have expanded the Discussion, giving a better representation of the value of the anesthetized data used for this study. I would accept this solution. However, the question remains whether "IO synchrony is indeed modulated during different phases of motor adaptation and learning" (see my remarks during the previous round). I do not feel that the new Fig. S7 helps to address this.

In other words, this study argues that IO dimensionality is related to IO synchrony (via gap junctions), and I agree with these conclusions, but leave me with the question to what extent this happens during real life. I would like to encourage the authors to address the expected variation in synchrony more explicitly.

We would like to thank the reviewer for making this important point more clearly as well as very encouraging words. We conducted a new analysis to address the reviewer’s concern. Specifically, we divided the complex spike data of 9 animals in the control condition into a series of shorter segments using a moving window, whose length varied in the range of [5, 50] seconds and the increment was 1 second. We next computed the dimensionality and the synchrony (both in 10 milli-seconds timescale) for each segment (panel A in the below figure) and then measured the correlation coefficient between these two metrics (panel B). As can be seen in panel C, the correlation coefficients were negative for almost all animals regardless of the segment length. These results provide clear evidence that synchrony variations in a physiological range are negatively correlated with changes in dimensionality, because the synchrony levels in the control complex spike data in our study are within the range of physiologically occurring synchrony levels in awake animals (Lang et al., 1999). During different phases of motor adaptation and learning, IO synchrony would likely be higher and as a consequence the correlations would become stronger. In the revised manuscript, we replaced the Poisson simulations in the figure S7, which seems to be obvious from the computational viewpoint, by this analysis and discussed this point in the text (Line 277-283).

*Figure legend: A: time-varying synchrony (left ordinate) and dimensionality (right ordinate) computed in a moving window of 10 seconds for the control animal #3. B: the correlation analysis between those two measures shown in A indicated a significant negative coefficient r = -0.6. C: the synchrony vs. dimensionality correlation coefficients for 9 control animals with the window length varied from 5 to 50 seconds.*
